# Supplementary figures and images for: Discrete GPCR-triggered endocytic modes enable β-arrestins to flexibly regulate cell signaling
Source: eLife. 2022 Oct 17;11:e81563. doi: 10.7554/eLife.81563 (PMC9681205; doi:10.7554/eLife.81563)

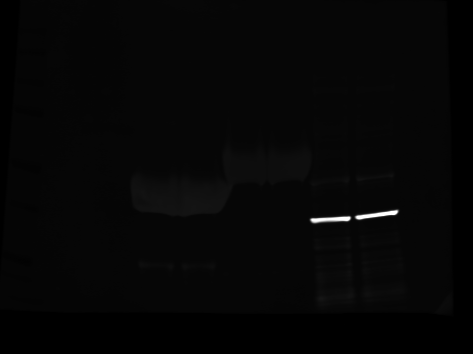

Supplement: Figure 1—figure supplement 1—source data 1. [file elife-81563-fig1-figsupp1-data1.zip › Figure 1-figure supplement 1-source data/20220921d_GST-CHC_GST-AP2_arr3_372T_pulldown-800.tif]

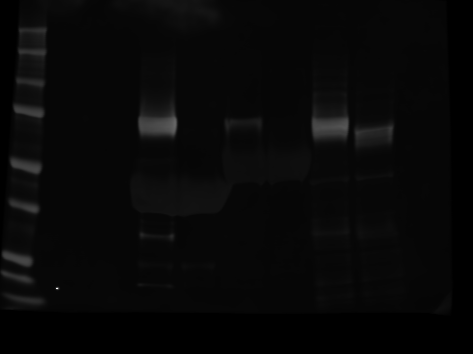

Supplement: Figure 1—figure supplement 1—source data 1. [file elife-81563-fig1-figsupp1-data1.zip › Figure 1-figure supplement 1-source data/20220921d_GST-CHC_GST-AP2_arr3_372T_pulldown-700.tif]
